# Stock Market Fluctuations and Self-Harm among Children and Adolescents in Hong Kong

**DOI:** 10.3390/ijerph14060623

**Published:** 2017-06-09

**Authors:** Wilfred Hing-Sang Wong, James Chun-Yin Lee, Frederick Ka-Wing Ho, Tim Man-Ho Li, Patrick Ip, Chun-Bong Chow

**Affiliations:** Department of Paediatrics and Adolescent Medicine, Li Ka Shing Faculty of Medicine, The University of Hong Kong, Hong Kong, China; whswong@hku.hk (W.H.-S.W.); jacylee@hku.hk (J.C.-Y.L.); fredkho@connect.hku.hk (F.K.-W.H.); timlmh@hku.hk (T.M.-H.L.); patricip@hku.hk (P.I.)

**Keywords:** self-harm, stock market, Hong Kong children

## Abstract

Although a few studies investigated the impact of stock market fluctuations on population health, the question of whether stock market fluctuations have an impact on self-harm in children and adolescents remain unanswered. This study therefore investigated the association between stock market fluctuations and self-harm among children and adolescents in Hong Kong. Daily self-harm attendance records were retrieved from all 18 local Accident and Emergency Departments (AED) from 2001 to 2012. 4931 children and adolescents who committed self-harm were included. The results indicated positive correlation between daily change in stock market index, Hang Seng Index (∇HSI, per 300 points), and daily self-harm incident risk of children and adolescents, without time lag between the two. The incident risk ratio for ∇HSI was 1.09 (*p* = 0.0339) in children and 1.06 (*p* = 0.0246) in adolescents. Importantly, non-trading days were found to impose significant protective effect in both groups against self-harm risk. Our results showed that stock market fluctuations were related to self-harm behaviors in children and adolescents. Parents and professionals should be educated about the potential harm of stock market fluctuations and the importance of effective parenting in reducing self-harm among children and adolescents.

## 1. Introduction

Self-harm, defined in this study as intentionally hurting one’s body or body tissue with or without suicidal intent [1], is one of the leading causes of morbidity among young people [2,3]. Children and adolescents are more vulnerable to self-harm compared to individuals from other age groups [4]. In Hong Kong, 32.7% of secondary one students attempted at least one type of self-harm [5], which is comparable to the alarming self-harm rate of 12–37% in the United States [6,7,8]. To better tackle this worldwide public health challenge, apart from analyses at the individual and family level, it would also be important to identify risk factors at the system level. Topics on macroeconomic influences have attracted growing research attention. Previous research found that stock market fluctuations had significant influences on population health [9,10]. Specifically, Cotti, Dunn, and Tefft (2015) found that stock market declines were associated with worse self-reported mental health and riskier health behaviors such as more frequent tobacco and alcohol use [10]. However, these findings mainly concern adults and primarily come from western populations which may not be generalizable to other populations. To examine macroeconomic influences on Chinese populations, Hong Kong provides a good setting for economic research of this kind because Hong Kong is a major international financial center and its citizens have a high participation rate in the stock market which is one of the highest in the world [11,12,13]. It is therefore reasonable to expect that results using Hong Kong financial data with population health data might generate new insights into macroeconomic influences on health.

Despite limited evidence specific to the effect of stock market on children, numerous studies (i.e., [14,15,16]) have demonstrated the vital role of economic phenomena in affecting self-harm behaviors at different ages. Following economic phenomena such as market plunge, higher levels of psychological distress were observed in investors [9]. For those who also hold a parent role, intense stress may reduce their ability to provide quality parenting. Children born to financially stressful families have been found to exhibit more behavioral difficulties as a result of poor parenting [17]. Research also shows that problematic behaviors including self-harm are closely associated with different family problems such as lack of communication with parents, poor familial relationships and bonding, increased family conflicts, and child abuse [5,14]. These problems are common in low-income families, especially those investing most of their saving in the stock market. It is possible that macroeconomic events trigger financial concerns which could render parents of these families to spend more time in analyzing stock market information. Preoccupation with financial issues in turn might lead to neglectful parenting behaviors [18]. As stated in Bowlby’s attachment theory [19], neglected children are more likely to report stress, isolation, and loneliness. A previous study found that decreased parental nurturance and inconsistent parental discipline were associated with depression and loneliness among adolescent daughters [20]. These children and adolescents may in turn use risky behaviors such as self-harm to escape from the negative emotions [21] or to seek sensation when there is not enough stimulation at home and in school [22].

It is also noteworthy that stock trading behaviors can be categorized into two domains, financial investment and gambling. The former is more rational with careful analysis of the market and different possible outcomes [23], whereas the latter involves sensation seeking and is subject to desire thinking [24,25]. It has been suggested that Chinese investors have high propensities in risk-taking characterized by high investment to income and assets ratios with weak-to-moderate risk bearing ability [23]. These investment behaviors resemble gambling behaviors. While most studies indicated economic crisis as a predictor of poor health outcomes [9,10,26,27], the gambling nature of Chinese stock markets may make it different from other markets, resulting in different patterns of health outcomes. Based on the principles of classical and operant conditioning [28], intermittent rewards, such as stock market rise which cannot be easily determined by the investor, could increase the urge of gamblers to return to the gambling activity. As gamblers tend to focus on positive consequences [29], gains from stock investment coupled with high preference for stimulation may drive some Chinese parents to take more risks with increased time spent in stock market. With its complex mechanisms, fluctuations in stock market—regardless of loss or gain—warrant further research investigations in order to enhance our understanding of its impact on not only adult investors themselves but also on their family members such as children and adolescents.

Using a social perspective, fluctuations in the stock market could also impact the affective state of market participants which constitutes the public mood state. Negative social cues such as having observed the negative consequences of excessive financial gambling are particularly influential to children and adolescents [30]. Research showed that social environment had significant influences on individual self-harm behaviors [31,32,33]. Adolescents who engage in self-harm behaviors tend to have less tolerance for distress and poor verbal skills [34]. It is possible that after stock market fluctuations, public anxiety rises due to increased economic uncertainties. With massive media coverage, these uncertainties may grow and indirectly increase self-harm behaviors in children and adolescents through inducing a negative public mood state. On the other hand, if our hypothesized effect of stock trading is true and significant, such an effect should dissipate on non-trading days such as public holidays and Sundays. Examination of such holiday effects would help unveil the significance and true effect of stock market activities on population health.

Despite previous studies [9,10,26,27], it remains unclear whether stock market fluctuations also play a role in the health-related behaviors of children and adolescents. The present study therefore aimed to examine stock market fluctuations as a predictor of self-harm behaviors among children and adolescents. The effect of holidays considered here as non-trading days including public holidays, Saturdays, and Sundays were also explored. We hypothesized that daily stock market fluctuations were associated with daily self-harm attendance rates of children and adolescents in Hong Kong and that the strength of this association was reduced during holidays.

## 2. Methods

### 2.1. Data

The primary outcome of this study was the rate of daily self-harm attendance at the Accident and Emergency Department (AED) in all Hong Kong public hospitals among subjects between 5–19 years old. Data on daily self-harm attendance rate was retrieved from the electronic database of Hong Kong Hospital Authority from 1 January 2001 to 31 December 2012. When the patients arrived at the AED, they were assessed and categorized into traumatic and non-traumatic cases by an experienced nurse. Then, traumatic cases would be further classified into following categories: common assault, indecent assault, child abuse, self-harm, traffic, industrial, and domestic and sports injury. Ambiguous cases would be considered unclassified. The date of AED attendance was recorded, and the demographic characteristics of the subjects including date of birth and gender were also collected. Ages of the subjects were calculated by subtracting date of birth from date of attendance which were in unit of days, then divided by 365 and rounded down to the nearest integer. Only cases that met the following criteria (i) aged between 5 and 19; and (ii) were classified into “self-harm” under the traumatic cases were included for further analysis. As mentioned earlier, self-harm was broadly defined in this study as intentionally hurting one’s body or body tissue with or without suicidal intent.

Hang Seng Index (HSI) is a composite index with high variation based on the market performance of major blue-chip stocks in the Hong Kong stock market. The 50 constituent stocks in HSI maintain an aggregate market value of about 60% of the total market value. HSI, which tracks the daily changes in stock market performance in Hong Kong, was the main predictor variable in this study. Information about the historical daily close prices of HSI, a time series variable, was retrieved from Yahoo! Finance for the time period between 2001 and 2012. Demographic information, namely the mid-year population sizes of age 5–19 during 2001 to 2012, was obtained from the Census and Statistics Department for adjustment in our proposed statistical model as an offset parameter.

### 2.2. Data Analysis

The sample was first categorized according to the age into two groups: 5–12 years old (children group) and 13–19 years old (adolescent group). To examine the association between self-harm daily incidence rate and HSI in the two age groups, a negative binomial regression model was used in determining the significance of the coefficient (β) in Formula (1). In the present study, a negative binomial regression model was used instead of the Poisson model because there was an over-dispersion in the model. In general, negative binomial regression is similar to Poisson regression except that it adopts an extra Gamma distributed variable for the adjustment on over-dispersion [35]. This can avoid the standard error estimations from being biased downward. Since the absolute value of HSI would have an inflated autocorrelation among itself, a best fit ARIMA model was being estimated by statistical software for this time series. The fitted ARIMA suggested implementing first-order differencing to the HSI time series before entering it into the formula. Therefore, the relative change of the daily price of HSI (∇HSI) was utilized as the independent variable instead of the raw values of daily HSI. Due to the fact that the influence with one index point change in HSI is infinitesimally small, the parameter was fit to a scale of 300 for a better interpretation of the results. In addition, previous studies pointed out that holidays impose a significant effect on the mood of individuals and the likelihood of suicide behavior [36,37,38]. Since there would be no trading in the stock market on Saturday, Sunday, and public holidays, a holiday indicator variable was included in the model as one of the confounders. The indicator variable obtained a value of 1 if there were no transactions of stock on that day and obtained a value of 0 otherwise. A long-term trend was used as another potential confounder by introducing a continuous count variable for day elapsed ranging from 1 to 4383 (i.e., from 1 January 2001 to 31 December 2012). Meanwhile, monthly pattern was also adjusted by involving a variable ranging from 1 to 12 for the corresponding months (i.e., January to December). Log of mid-year population sizes, log (N), of 5–12 and 13–19 age groups for each year from 2001 to 2012 were used as an offset in the two respective equations correspondingly to adjust for the changes in population figures.

(1) $$\log\left(E\left[Y_{t}\right]\right)=\alpha +\beta \left(\nabla HSI\right)+\gamma_{1}\left(Holiday\right)+\gamma_{2}\left(t_{long}\right)+\gamma_{3}\left(t_{season}\right)+\log\left(N\right)+\varepsilon -\left(1\right)$$

In the model, E[Y_t_] is the expected number of self-harm AED attendance in Hong Kong in day *t*. Generally, the exponential of the regression coefficient (β) in the negative binomial regression model refers to incidence risk ratio, which captures the multiplicative effect of the independent variable on the response variable. In this model, significance of β would indicate that there is a multiplicative effect on the daily change of HSI towards the adjusted risk in the self-harm attendance number. All results were presented on an incident risk ratio (IRR: exp(β)). Results of the analyses with a *p*-value less than 0.05 would be considered as statistically significant. All statistical computations were done by statistical software R (R for Windows, version 3.2.4).

## 3. Ethics

The ethics of this study were approved by the institutional review board of the University of Hong Kong—the Hospital Authority Hong Kong West Cluster, reference number: UW 12-495.

## 4. Results

A total of 4931 subjects aged 5–19 attended AED in Hong Kong public hospitals during 2001 to 2012 because of self-harm. The total number of self-harm attendance and its related incidence rate was presented in Table 1. The results demonstrated gradual decreases of absolute count in the number of self-harm attendance and incidence rate for both age groups throughout the years. The value of daily change in HSI varied from a minimum of −2061 points to a maximum of 2333 points over a 12-year period.

The negative binomial regression model found a positive association between first-order difference HSI and daily self-harm AED attendance rate in children and adolescents. There was a significant IRR of 1.09 for the children group (*p* = 0.0339) (Table 2). In contrast, the IRR for the adolescent group also yielded a significant positive result but with a slightly lower estimation of 1.06 (*p* = 0.0246) (Table 2). For every 300 HSI points increment in the closing price relative to the previous trading day’s closing price, there was an increase in self-harm rate by a factor of 1.09 in the children group and 1.06 in the adolescent group provided that other variables remained constant. In other words, a daily increment in the stock market index was associated with an increase in the daily self-harm AED attendance. On the other hand, the non-trading day indicator variable was found to impose significant protective effect with a factor of 0.799 (*p* = 0.0003) for the children group and 0.849 (*p* = 0.0001) for the adolescent group. As expected, the self-harm incidence rates were higher among children and adolescents during stock market trading days compared with non-trading days.

## 5. Discussion

To the best of our knowledge, this is the first study examining the association between stock market fluctuations and self-harm rate of children and adolescents. The empirical results showed that stock market fluctuations were significantly associated with the self-harm behaviors in children and adolescents. In general, a daily increment of 300 HSI points was associated with an increased risk of daily self-harm by 9% and 6% for the children and adolescent groups, respectively. Younger subjects seem to be more vulnerable to the effect of stock market fluctuations. Furthermore, self-harm rates were also lower on non-trading days.

Stock market fluctuations can be contributed by various factors including those related to the intrinsic business performance and balance between supply and demand of individual stock companies, and external factors related to government policies and global economic environment [39]. Unlike major western stock exchanges like New York and London that are dominated by fund investors, there is comparatively higher participation of individual investors who invest directly in the Hong Kong Stock Exchange. In Hong Kong, individual stock investors constituted about 16% of the adult population, or 841,000 individuals, in 1999 but the number increased drastically to 35.7% or about 2.1 million adults in 2011 [18,19]. There was an approximately 2.5 times increase in the number of investors within the 12 years, signifying the escalating trend of stock market participation in this financial center in southern China. In view of the increased pool of stock market participants among Hong Kong population, the influences of fluctuations in the stock market, which was represented by HSI in this study, could be widely spread and affect the public mood state.

Although children and adolescents are less likely to participate in stock market trading, their psychosocial wellbeing could easily be affected by the public mood state, which is closely tied to the emotions of parents who have participation in stock market trading, especially during trading days. The findings of this study are compatible with the family attachment theory [40,41] in that reduced parent–child interactions as a result of engagement in stock trading could contribute to insecure attachment between children and caregiver and weak family cohesion which were known risk factors for self-harm in children [42]. On the other hand, during non-trading period, parents may be less involved and pay less attention on stock trading which in turn allows them to spend more time with their children. This indirect effect of non-trading days could benefit children’s wellbeing and reduce their self-harm risk.

A particularly interesting result of this study is that increases in stock price predicted higher self-harm rate among children and adolescents. This is an innovative finding because most literature suggests that stock market plunge was associated with poor health outcomes of individual investors [9,10,26]. Also in line with our finding of holiday effect, some research found that economic downturns exert health-protective effects on children [43]. Although different from the normal belief, using gambling concepts could shed light on this new observation. It has been suggested that Chinese investors are mostly risk takers who often overestimate their chance of success without analyzing possible outcomes. They mainly rely on informal means such as newspaper to make investment decisions without having adequate stock trading knowledge [23]. According to this speculation, when stock market rises, parents with high preference for sensation who are also market participants might have a stronger urge to invest as a result of reinforcement by the positive feedback from stock market. This can lead to further reduction in the amount of time parents spend with their children. Consequently, these children may experience greater loneliness and may use riskier behaviors such as self-harm to cope with the negative feelings toward the society. It may also explain why we found younger children who still relied on parents to provide nurturance and care were more vulnerable to stock market fluctuations.

Together with previous research findings, our results provide additional support for the impacts of stock market fluctuations, regardless of loss or gain, on a society. Moreover, we strengthened the evidence by demonstrating that the stock market impacts were reduced on non-trading days. Despite the knowledge added, the following limitations should be considered when interpreting the findings of the current study. First, results of this study did not adjust for the potential confounding effects of some important individual factors such as family functioning, parental mental health, parenting styles, and children’s risky behavior. Past studies showed that adolescents who came from disadvantaged families with more parental conflicts and weaker communication engaged in more self-harm behaviors [44,45,46]. Meanwhile, substance abuse, including tobacco and alcohol use could also increase the likelihood of self-harm [43,47]. However, it remains uncertain whether the relationship between stock market fluctuations and self-harm among children and adolescents is confounded by the above listed factors and such question should be followed up in future investigations. Secondly, this study only examined the association between stock market fluctuations and self-harm AED attendance rate, yet a causal relationship could not be established due to the nature of study design and data analysis. Third, the cases of self-harm in this study only included those attending AED in public hospitals and this may not capture all self-harm cases in Hong Kong. Some mild self-harm cases like minor skin cutting and burning were managed at outpatient clinics and hence did not result in any AED attendance; as for severe self-harm cases, they may result in death and may be considered suicide incidents. These missing self-harm cases may affect the analysis in this study. Fourth, the current study did not examine the potential mechanisms underlying the association of stock market fluctuations with self-harm risk among children and adolescents. Further investigations are needed to elucidate these underlying mechanisms.

## 6. Conclusions

To conclude, stock market fluctuations were associated with self-harm behaviors among children and adolescents in Hong Kong. In particular, young children seem more vulnerable to the stock market changes. Parents’ excessive involvement in stock market trading might lead to neglectful parenting, which in turn could give rise to a higher risk of self-harm in their children. Parents and professionals should pay extra attention to the potential harm of stock market performance on individual investor’s emotions, which could indirectly influence the wellbeing of children and adolescents in their families. Parents should also be educated about the importance of effective parenting in reducing children’s self-harm. Further investigations are needed to delineate the underlying mechanisms of the association between stock market fluctuations and self-harm behaviors in children and adolescents.

## Figures and Tables

**Table 1:** Self-harm attendance number and corresponding attendance rate per 100,000 persons.

Year	Age 5 to 12		Age 13 to 19	
	Total No. of Self-Harm	Incidence Rate per 100,000 Persons	Total No. of Self-Harm	Incidence Rate per 100,000 Persons
2001	530	80	529	85
2002	254	39	416	67
2003	230	36	276	45
2004	121	20	287	47
2005	87	15	223	36
2006	156	27	277	45
2007	125	23	276	45
2008	88	17	212	34
2009	44	9	174	28
2010	26	5	139	23
2011	75	17	191	33
2012	32	7	163	29
Total	1768		3163	

**Table 2:** Estimated Coefficients in Negative Binomial Regression of (**a**) Children (Age 5 to 12) and (**b**) Adolescents (Age 13 to 19).

	(a) Age 5 to 12		(b) Age 13 to 19	
	Estimate	95% CI	Estimate	95% CI
∇HSI	0.0864 *	(0.0066, 0.1662)	0.0570 *	(0.0073, 0.1067)
Holiday	−0.2248 **	(−0.3467, −0.1029)	−0.1632 **	(−0.2459, −0.0805)
t_long_	−0.00052 **	(−0.00057, −0.00047)	−0.00027 **	(−0.0003, −0.00024)
**January (Baseline)**				
February	−0.3158 *	(−0.5723, −0.0593)	0.0619	(−0.1254, 0.2492)
March	−0.1894	(−0.4335, 0.0548)	0.1808 *	(0.0020, 0.3596)
April	−0.3347 *	(−0.5895, −0.0800)	−0.0500	(−0.2399, 0.1399)
May	−0.2743 *	(−0.5243, −0.0243)	0.1842 *	(0.0048, 0.3636)
June	−0.4502 **	(−0.7133, −0.1871)	0.0320	(−0.1549, 0.2189)
July	−0.3371 **	(−0.5920, −0.0823)	−0.0082	(−0.1956, 0.1792)
August	−0.2818 *	(−0.5345, −0.0292)	0.0486	(−0.1368, 0.2340)
September	−0.3522 **	(−0.6124, −0.0921)	0.1983 *	(0.0169, 0.3797)
October	−0.1010	(−0.3463, 0.1442)	0.3342 **	(0.1586, 0.5098)
November	−0.2238	(−0.4782, 0.0306)	0.1434	(−0.0407, 0.3275)
December	−0.5328 **	(−0.8054, −0.2601)	−0.1612	(−0.3578, 0.0354)

Significance codes: * *p* < 0.05, ** *p* < 0.01.
